# The Neurovascular Niche: A Gathering Venue for Neuroinflammation and Remyelination in Multiple Sclerosis

**DOI:** 10.1111/apha.70285

**Published:** 2026-08-12

**Authors:** Ilias Kazanis, Maria Elena Silva, Alerie G. de la Fuente, Fernando de Castro, Francisco J. Rivera

**Affiliations:** ^1^ Centre for Resilience, Ageing Biology and Age‐related Diseases Group, School of Life Sciences University of Westminster London UK; ^2^ Translational Regenerative Neurobiology Group (TReN), Department of Molecular and Integrative Biosciences (MIBS), Faculty of Biological and Environmental Sciences University of Helsinki Helsinki Finland; ^3^ Instituto de Neurosciencias CSIC‐UMH, San Juan de Alicante Alicante Spain; ^4^ Myelin Dynamics & Repair Lab, Centro de Neurociencias Cajal/Instituto Cajal‐CSIC Alcalá de Henares (Madrid) Spain

**Keywords:** blood elements, CNS stem/progenitor cells, CNS vascular and perivascular cells, immune cells, Multiple sclerosis, neuroinflammation, remyelination

## Abstract

In the central nervous system (CNS), the tissue microenvironment is continuously monitored and regulated to secure the unobstructed function of neurons and of their networks. This is a key function of the neurovascular niche (NVN), which is the interface between the cells of the nervous tissue and the cells and the content of blood vessels. It is enabled by the Blood–Brain Barrier, a structure formed by endothelial and perivascular cells, extracellular matrix, and astrocytes, and is manifested by the limited surveillance of the CNS from blood‐derived cells. Multiple sclerosis (MS) is a devastating degenerative disorder, in which the myelin sheaths that enwrap neuronal axons are destroyed, leading, over time, to neurological symptoms. MS has a strong immunological component which is targeted in most of the current disease‐modifying treatments. Nevertheless, regenerative interventions aiming at enhancing and restoring the endogenous remyelination potential of the CNS, driven by the abundant Oligodendrocyte Progenitor Cells (OPCs), have not been successfully developed so far. Here, we will review key information on the structure of the NVN, and we will summarize the evidence on the role of inflammation in the emergence and the progress of MS, with a focus on the active response of OPCs. We will also present recent experimental evidence on the role of less investigated cellular elements of the NVN, such as pericytes and platelets, in the regulation of OPCs. Finally, we will discuss current and future treatments for MS.

## 1 Introduction

### 1.1 Remyelination Failure in Multiple Sclerosis: Mechanisms and Therapeutic Challenges With a Focus on Oligodendrocyte Progenitors

Multiple sclerosis (MS) is the most common neurological disease among young adults. It is an autoimmune‐mediated demyelinating disease of the central nervous system (CNS), affecting about 2.8 million patients worldwide [1]. The cause of MS, as well as the reasons for its variation among patients, remains elusive. Current treatments aimed at preventing or inhibiting the autoimmune aspect of the disease have led to significant clinical successes but show limited effectiveness in the progressive forms of MS; they have side effects, such as intracerebral hemorrhages [2] and fail to promote the regeneration of lost myelin (reviewed in [3, 4, 5]). In the healthy brain, remyelination represents the regenerative response to myelin damage and is robust and efficient [6, 7]. This process is facilitated by the presence of CNS‐resident oligodendrocyte progenitor cells (OPCs), which are widely distributed throughout white and gray matter. In response to demyelination, OPCs become activated, proliferate, and are recruited to the lesion site, where they differentiate into remyelinating oligodendrocytes (reviewed in [6, 7]). In addition to OPCs, neural stem/progenitor cells (NSPCs) located in the subependymal zone niche (SEZ) (also known as the ventricular‐ subventricular zone), found adjacent to the walls of the lateral ventricles, serve as an alternative source of newly generated oligodendrocytes in response to myelin damage, particularly in the corpus callosum (CC) [8, 9]. However, unlike CC‐resident OPCs, SEZ‐derived NSPCs fail to give rise to properly myelinating oligodendrocytes [10]. Notably, remyelination fails, especially as a function of age, extensively in MS. Therefore, identifying and elucidating the cellular and molecular cues and mechanisms that control OPC function and remyelination, as well as understanding the reasons for its failure in MS, will be a critical milestone in this research field, essential for developing regenerative strategies.

### 1.2 The Neurovascular Niche: Cellular and Molecular Components

**FIGURE 1:**
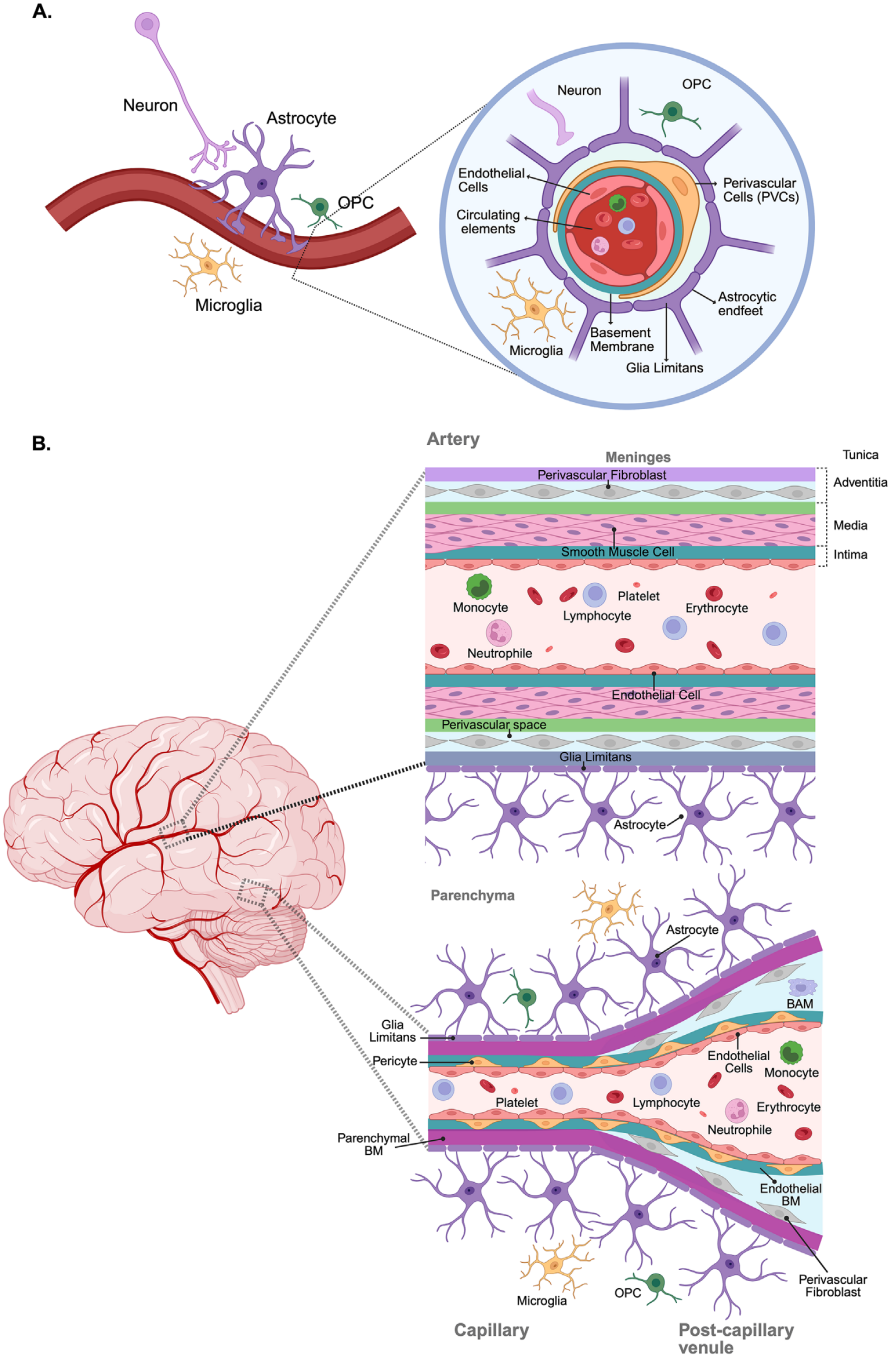
The neurovascular Niche in the CNS. Illustrations of the different components of the neurovascular niche (NVN) in the CNS. (A) Image illustrating a generic blood vessel and surroundings cells in the CNS, with a transverse zoomed view (cross‐section) showing the components of the NVN: Endothelial cells, circulating elements, basement membrane, perivascular space, perivascular cells, astrocytic endfeet, microglia, oligodendrocyte progenitor cells (OPCs) and neurons. (B) Image illustrating the brain with two zoomed views: An upper panel shows an arterial vessel, while the lower panel displays the continuum of microvessels/capillaries leading to a post‐capillary venule. Upper panel: In the CNS, arteries consist of endothelial cells and a three‐layered structure (tunica intima, media and adventitia) with vascular smooth muscle cells (vSMCs) and perivascular fibroblast (PVFs) continuum. These CNS large vessels have a perivascular space and a glia limitans. Circulating elements include erythrocytes, monocytes, lymphocytes, neutrophiles, platelets, etc. Lower Panel: Pericytes (PCs) are located within the inner basement membrane along pre‐capillaries and capillaries, while perivascular fibroblasts (PVFs) primarily reside between the endothelial base membrane and the outer parenchymal basement membrane, specifically within the perivascular space along post‐capillary venules. This perivascular space may also contain border‐associated macrophages (BAM). Additionally, astrocytes extend processes to form endfeet, contributing to glia limitans perivascularis. Capillaries lack perivascular space. Note the proximity of OPCs and microglia cells near the glia limitans. Illustrations created by BioRender.

The neurovascular niche (NVN) in the adult CNS consists of a diverse array of cells, including those that contribute to the vascular structure, those located within the perivascular parenchyma, and cellular elements circulating in the blood [11, 12, 13] (Figure 1A). The vascular component comprises endothelial cells, the basement membrane, and associated perivascular cells (PVCs) (Figure 1A,B). PVCs include mural cells (integral components of the vascular wall), such as vascular smooth muscle cells (vSMCs) and pericytes (PCs), as well as cells outside the mural cell layer, separated by an endothelial basement membrane, such as perivascular fibroblasts (PVFs) [14, 15, 16, 17]. Vessel composition varies with caliber: large vessels have distinct tunica layers, capillaries lack a perivascular space, and the composition of PVCs differs across large (arteries and veins), medium (arterioles and venules), and small vessels or capillaries (Figure 1B). vSMCs and PVFs localize to arteries, arterioles, venules, and veins, while PCs are restricted to capillaries or microvessels [18, 19, 20, 21] (Figure 1B).

The perivascular parenchymal component of the NVN consists of CNS‐resident cells located near PVCs, including astrocytic processes and their endfeet, which contribute to the formation of the glia limitans perivascularis, as well as microglia (reviewed in [20]) (Figure 1A,B). Additionally, intrinsic spatial and functional interactions between blood vessels and CNS progenitor cells—mainly OPCs—have been identified [22, 23], especially during cell migration [24, 25]. Neurons are also located near perivascular areas (reviewed in [26, 27]) (Figure 1A).

Finally, circulating cells represent an essential component of the NVN, including red blood cells, platelets, monocytes/macrophages, neutrophils, and lymphocytes (reviewed in [12]) (Figure 1A,B). Overall, the NVN serves as a specialized microenvironment where various cellular and extracellular components of blood vessels and the circulating elements interact not only with each other but also with surrounding CNS‐resident cells.

### 1.3 Neurovascular Niche‐Neuroinflammation Crosstalk: Implications for Remyelination in MS

The blood–brain barrier (BBB), which characterizes the CNS vasculature, is formed as a result of interactions within the NVN to control the molecular and cellular exchange between the circulation and the CNS. The BBB is composed of endothelial cells, the basement membrane, perivascular cells, the glia limitans, and microglia (reviewed in [20, 27]). Notably, in MS breakages of the BBB occur, allowing encephalitogenic T and B cells, macrophages, and other immune cells to infiltrate the brain and spinal cord. This is accompanied by the activation of CNS‐resident microglia and astrocytes, contributing to demyelination and, ultimately, axonal loss (reviewed in [5, 20]). The current hypothesis is that during neuroinflammation, the cytoarchitecture of the NVN is damaged, with its altered organization contributing to disease progression.

Do the components of the NVN modulate neuroinflammation in response to myelin damage? Do they contribute to remyelination and its failure in MS? Evidence suggests that they do. Several studies indicate that NVN elements play an essential role in modulating neuroinflammation and OPC function during remyelination. For example, upon demyelination, macrophages are recruited from the bloodstream to the lesion site, where they transit through various inflammatory stages, promoting myelin debris clearance and facilitating OPC differentiation to support remyelination [28, 29, 30]. Additionally, different studies have demonstrated that other circulating factors, such as T cells and platelets, are recruited to demyelinating areas, where they modulate OPC differentiation and contribute to remyelination [31, 32, 33]. The role of the NVN in remyelination is not limited to blood elements; in response to myelin damage, vascular pericytes proliferate and secrete soluble factors that support OPC differentiation during remyelination [23]. Thus, there is evidence indicating a driver role of the NVN to remyelination. Moreover, various studies suggest that alterations in NVN components observed in MS may contribute to a dysregulation of neuroinflammation and remyelination failure in this disease. For instance, perivascular cells and platelets exhibit abnormalities in MS [34, 35, 36, 37], which affect their functions and may consequently hinder their role in remyelination. Additionally, OPC function is not limited to the generation of new myelinating oligodendrocytes; these cells may display an active immune role (see next chapter).

In summary, there is substantial evidence that positions the NVN as a critical CNS microenvironment enabling the crosstalk between blood vessels, circulating blood, and immune cells, as well as OPCs. This is essential for the neuroinflammatory response as well as for proper and effective remyelination. In this review, we will focus on OPCs, comprehensively summarizing emerging evidence showing that these cells not only pathetically suffer the consequences of neuroinflammation, but also actively contribute to the response of the tissue. We will also highlight findings revealing how different cellular components of the NVN interact with OPCs during MS, contributing to remyelination failure. Finally, we discuss current NVN‐targeted experimental therapies for the treatment of MS.

## 2 The Neuroinflammatory Microenvironment as a Cause of MS

Neuroinflammation has been directly associated with MS and, indirectly, with other demyelinating diseases such as neuromyelitis optica. In MS, neuroinflammation is characterized by dysregulated cytokine, chemokine, and intracellular signaling pathways (such as that of NF‐κB), as well as by the activation of the inflammasome, which orchestrates the recruitment and activation of immune cells within the CNS. Resident cells, such as microglia and astrocytes, together with the infiltrating peripheral immune cells, create a microenvironment that promotes demyelination and hampers remyelination, leading to chronic gliosis and progressive axonal degeneration [6, 7]. MS has been traditionally considered to be an autoimmune demyelinating disease, although a growing bulk of evidence is forcing scientists to substitute this view with that of a complex demyelinating disease with neurodegenerative‐plus‐autoimmune pathogenic components [3, 38, 39], partly because the etiology of the death of oligodendrocytes and of the subsequent loss of myelin is still unknown (for a recent review, see [40]). It is true that the most frequent clinical form of MS is the Relapsing–Remitting one (RR‐MS), in which the sudden appearance of relapses of disability is linked to identifiable inflammatory processes. Over time, symptoms remit with patients recovering to a basal state. Nevertheless, after an undefined number of relapses, neurological symptoms, underlined by demyelination, start, leading to cumulative neurological damage [41, 42]. Approximately 50% of RR‐MS cases, even those successfully treated with disease‐modifying treatments (see Table 1 for a review of the current available treatments of MS), evolve into a secondary progressive neurodegenerative phase (SP‐MS). SP‐MS is characterized by an accumulation of neurodegenerative symptoms and an absence of relapses [41]. Around 15% of MS patients exhibit progressive neurodegeneration post‐diagnosis, with no apparent inflammatory relapses [39, 41]. This phenomenon is often referred to as “progression independent of relapse activity” and accumulating data suggest that it is caused by smoldering inflammation [79], which could be triggered by demyelination or energy deficiency. These cases present a significant clinical challenge in terms of their assessment, sometimes associated with gray matter atrophy and diffuse microglial activation, distinct from focal neuroinflammatory incidents [3, 39, 79, 80]. In a very low number of cases, only a single inflammation‐linked relapse event appears in the absence of any new events, even for decades, showing very mild progress. This form has been defined as clinically isolated symptom (CIS) or benign MS [41]. Therefore, even though we still do not know the initial cause(s) of the disease, inflammation (visible or not) plays a role in MS progress and in the sequence of the disease's stages [39, 79]. Notably, the immune system's response to oligodendroglial cell death and to myelin debris seems to be of capital importance [3, 81]. Cytokines (such as IL1beta, IL6, TNF, CCL2, GM‐CSF) released by microglia and activated astrocytes, together with reactive oxygen species (ROS) (normally generated by microglia/macrophages), do not only perpetuate damage to oligodendrocytes and demyelination but also trigger neuronal damage [3]. Excitotoxicity, oxidative stress, and mitochondrial injury are the main mechanisms responsible for axonal loss, synaptopathy, and finally, neuronal death in chronic smoldering inflammation and in the late stages of MS [3, 82], while ferroptosis emerges as a common mechanism inducing the death of neurons and oligodendrocytes [82, 83]. High‐throughput studies are unveiling the different roles of the many cell types that are involved in demyelination, including a protective effect of the inflammatory reaction, fully incorporating them into the global overview of demyelinating damage [84]. For example, myeloid‐derived suppressor cells (MDSCs), a population of innate immune cells, have been shown not only to limit the initial inflammatory reaction [85, 86, 87] but also to release neuroprotective and remyelinating cues like osteopontin [88, 89, 90]. Based on the above, MDSCs are a valid target for the design of cell therapy approaches for MS [91, 92].

**TABLE 1:** Treatments for multiple sclerosis and their impact on NVNs components: Commercial and under development.

Drug	Current status	Major therapeutic goal	Target any of the NVNs component	Mechanism of action	Refs^a^
Alemtuzumab	Commercial	Immunomodulation	Yes (B cells, T cells)	Anti‐CD52	[43]
Cladribine	Commercial	Immunomodulation	Yes (T cells, B cells)	Purine analog	[44]
Daclizumab	Commercial/withdrawn	Immunomodulation	Yes (T cells)	Anti‐CD25	*Withdrawn 2018* ^b^
Dimethyl Fumarate	Commercial	Immunomodulation, Neuroprotection	Yes (T cells, B cells)	Nrf2, antioxidant	[45]
Fampridine (dalfampirine, 4‐amynopiridine)	Commercial	Neuroprotective	No (Neurons) Not clear the effect on B & T cells	K^+^‐channels blocker	[46]
Fingolimod hydrochloride	Commercial	Immunomodulation	Yes (T cells, endothelial cells, B cells, OPCs?)	Sphingosine S1P_1–5_ receptors inhibitor	[47]
Glatiramer Acetate	Commercial	Immunomodulation	Yes (T cells)	Unclear	[48]
Interferon Beta‐1a	Commercial	Immunomodulation	Yes (T cells)	JAK–STAT activator	[49]
Interferon Beta‐1b	Commercial	Immunomodulation	Yes (T cells)	JAK–STAT activator	[50]
Mitoxantrone	Commercial	Immunosuppressive	Yes (T cells, B cells)	Cytotoxic	[51]
Natalizumab	Commercial	Immunomodulation	Yes (T cells, B cells)	Alpha‐Integrin inhibitor	[52]
Ocrelizumab	Commercial	Immunomodulation	Yes (B cells)	Anti‐CD20	[53]
Ofatunumab	Commercial	Immunomodulation	Yes (B cells)	Anti‐CD20	[54]
Peginterferon Beta‐1a	Commercial	Immunomodulation	Yes (T cells)	JAK–STAT activator	[55]
Ponesimod	Commercial	Immunomodulation	Yes (T cells, endothelial cells)	S1P_1_ receptor inhibitor	[56]
Rituximab	Commercial	Immunomodulation	Yes (B cells)	Anti‐CD20	[54]
Siponimod	Commercial	Immunomodulation	Yes (T cells, endothelial cells)	S1P_1_ & S1P_5_ receptors inhibitor	[57]
Teriflunomide	Commercial	Immunomodulation	Yes (T cells, B cells)	Unclear (inhibits dihydroorotate dehydrogenase?)	[58]
Ublituximab	Commercial	Immunomodulation	Yes (B cells)	Anti‐CD20	[54]
Benzotropine	Commercial (for Parkinson's disease and extrapyramidal reactions)	Remyelination	Yes (OPCs)	Histamine H1 receptor antagonist M1 muscarinic receptor antagonist, inhibits Dopamine reuptake	[59, 60]
CART‐T cells autologous transplantation	Under development	Immunomodulation	Yes (B cells)		[61]
BIIB033 (biological)	Abandoned	Remyelination	Yes (OPCs)	Anti‐LINGO‐1 blocking antibody	[62]
Clemastine	Commercial (repurposing)	Remyelination	Yes (OPCs)	Histamine H1 receptor blockade, Antimuscarinic	[63]
GSK239512	Under development	Remyelination (very poor)	Yes (OPCs)	Histamine H3 receptor blockade	[64, 65]
GNbAC1	Under development	Remyelination	Yes (OPCs)	Anti‐ENV humanized antibody	[66, 67]
Myaptavin‐3064	Under development	Remyelination	Yes (OPCs)	Aptamer conjugated	[68]
rHIgM22 (biological)	Under development	Remyelination	Yes (OPCs)	IgM22 human recombinant monoclonal autoantibody	[69]
TC3.6	Under development	Remyelination	Yes (OPCs)	PDE7 inhibition	[70, 71, 72]
TDZD8	Under development	Remyelination	Yes (OPCs)	GSK3 inhibition	[71, 73]
VP1.15, VP3.15	Under development	Remyelination	Yes (OPCs)	Dual PDE7/GSK3 inhibition	[71, 74, 75]
VX15/2503 (biological)	Under development	Remyelination	Yes (OPCs)	Humanized IgG4 anti‐Sema4D	[76]
ApTOLL	Under development	Immunomodulation, Neuroprotection, Remyelination	Yes (T cells, OPCs)	TLR4 receptor blockade	[77]
Bavisant	Under development	Neuroprotection, Remyelination	Yes (OPCs)	Histamine H3 receptor antagonist	[78]

^a^ Either original publication (for recent compounds) or actualized review (for commercially available ones).

^b^ https://www.ema.europa.eu/en/news/ema‐urgently‐reviewing‐multiple‐sclerosis‐medicine‐zinbryta‐following‐cases‐inflammatory‐brain‐disorders.

## 3 At the CNS Side: OPC‐Immune System Interaction Is Essential for Neuroinflammation and Remyelination in MS

The NVN constitutes a critical gathering venue for cells of the immune system and OPCs. In homeostatic conditions, it acts as a selective gateway, tightly regulating immune cell entry into the CNS. However, it becomes permissive under conditions of cellular stress or in diseases like MS [93]. This increased permissiveness in the NVN is mainly driven by BBB breakdown, and the increased expression of VCAM‐1, ICAM‐1, as well as chemoattractant cytokines and chemokines, such as CCL2 and CXCL10, by endothelial cells, which enable leukocyte adhesion and enhance transmigration (reviewed in [94]). The NVN also acts as a migration scaffold for OPCs. During development, OPCs migrate along blood vessels and once they reach their final destinations, they detach from the blood vessels to colonize the CNS parenchyma [22], a process also observed in adult, NSPC‐derived, OPCs [24]. In cases of white matter injury, this perivascular migration becomes aberrant due to the inability of OPCs to detach from blood vessels. These stationary OPCs contribute to the disruption of the BBB [95], enhancing further the inflammatory reaction.

**FIGURE 2:**
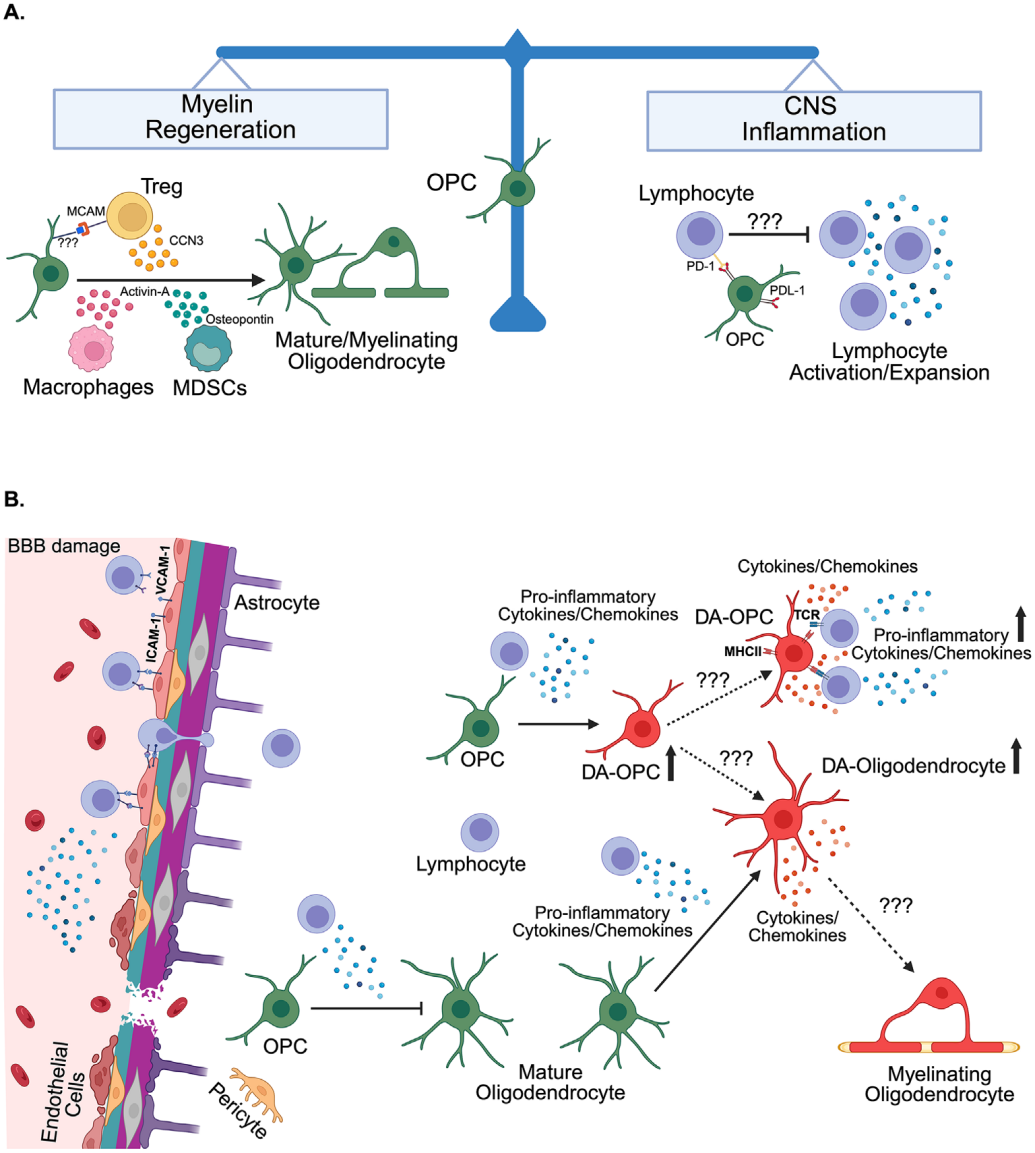
OPCs are active regulators of immune and regenerative processes in the CNS. (A) OPCs are increasingly recognized as key players at the intersection between inflammation and repair. As oligodendrocyte progenitors, they receive signals from other cells from the CNS microenvironment, mainly other immune cells which drive their differenatiation to mature oligodendrocytes. Additionally, OPCs can also upregulate the expression of immune regulatory molecules such as PD‐L1 and limit lymphocyte‐drive inflammation. (B) BBB damage leads to the detachment of pericytes from vessels and infiltration of lymphocytes in the CNS. Lymphocytes secrete pro‐inflammatory factors the drive the transition of OPCs and mature oligodendrocytes to a disease‐associated (DA) phenotype charaterised by the expression of antigen processing and presentation molecules and pro‐infoammatory cytokines and chemokines that may further perpetuate inflammation. However, the role of these disease‐associated OPCs and oligodendrocytes in remyelinations remains still a matter of debate. Images are done with BioRender.

Within the demyelinated white matter, oligodendrocyte lineage cells ‐both OPCs and mature oligodendrocytes‐ are frequently found in close proximity to immune cells of both innate and adaptive origin [32, 84, 88, 96, 97, 98]. Historically, OPCs were considered passive participants in neuroinflammation as their role was masked by the fact that the immune system is a major driver of CNS demyelination and neurodegeneration. Pro‐inflammatory lymphocytes such as Th1 and Th17 cells not only target mature oligodendrocytes causing demyelination [99, 100] but also impair OPC differentiation and thus limit remyelination. For example, lymphocytes isolated from peripheral mononuclear blood from MS patients inhibit remyelination when transplanted into a lysolecithin‐driven demyelination mouse model [101]. Similarly, CD4+ Th17 cells disrupt OPC proliferation and differentiation in a cuprizone model of de‐ and remyelination [102]. Paradoxically, the immune system also plays a critical protective role in myelin regeneration. In lysolecithin‐induced demyelination models, the absence of adaptive immunity (e.g., Rag1 knockout mice or T‐cell depletion model [103]) or the depletion of macrophages using clodronate liposomes [104], significantly impairs myelin regeneration. This regenerative effect is largely attributed to the pro‐regenerative role of regulatory T cells and anti‐inflammatory macrophages, which enhance OPC differentiation through direct cell to cell contact [32] and/or the secretion of pro‐regenerative factors such as cellular communication network factor 3 (CCN3) [31] or activin‐A [28] (Figure 2A).

However, the restricted view of OPCs only as receptors of immune signals has changed significantly in recent years, with this progenitor population being now considered to be an active modulator of the neuroinflammatory environment (Figure 2A). As stem‐like cells, OPCs act as sensors of inflammatory cues, responding rapidly to injury in both primary and secondary demyelination [105, 106]. These responses include morphological changes‐such as process shortening and thickening [107], reminiscent of the activation of microglia‐ and acquisition of a distinct transcriptomic profile characterized by immune‐related gene expression, termed disease‐associated OPC (DA‐OPC) phenotype [108, 109, 110] (Figure 2B). In a mouse model of immune‐mediated demyelination such as experimental autoimmune encephalomyelitis (EAE), DA‐OPCs emerge early in regions with sparse immune infiltration and in the vicinity (although not necessarily in direct contact) of vascular and immune cells and their number increases with the peak of inflammation and disease progression (Figure 2B). This suggests that DA‐OPCs may be induced by soluble inflammatory cytokines penetrating the CNS parenchyma [84]. Similar immune gene signature is also observed in OPCs derived from iPSCs of MS patients [111], and chromatin accessibility studies confirm that OPCs in MS patients and EAE are primed to rapidly activate immune pathways under inflammatory conditions [112, 113]. Whether DA‐OPCs represent a transient state in response inflammation or a stable population‐and their precise role in remyelination‐remains unresolved.

The expression of antigen processing and presentation machinery [108, 109, 114, 115], immunoproteasome components [96], cytokines and chemokines [114, 116], as well as of phagocytic machinery [96, 108, 117] in OPCs positions them as potential immunomodulators contributing to balance neuroinflammation and restore homeostasis. However, this role appears to be context‐dependent, varying in acute versus chronic inflammation with the potential of being both protective and detrimental to CNS health.

Under inflammatory conditions, OPCs upregulate the expression of cytokines and chemokines at both the mRNA and protein levels as well as of extracellular matrix proteins and related regulators (e.g., CCL2, IL1β, IL33, B2M, SERPINA3N), some of which may function as chemoattractants for immune cells [108, 116, 117, 118]. A recent pre‐print further suggests that some of these molecules may be secreted by OPCs, as they are detected in the OPC secretome following exposure to inflammatory cues such as TNF‐α and IFN‐γ [119]. Additionally, OPCs can express metalloprotein matrix 9 (MMP9), which is involved in blood‐brain barrier opening [120] and may thereby further contribute to CNS immune infiltration. Altogether, these data suggest an active role for OPCs in immune cell recruitment to the CNS during inflammation. This may aid adequate pathogen clearance during infections, having a protective role in this context. Once infections are cleared, OPCs may help resolve inflammation and restore CNS homeostasis, as suggested by different OPC‐depletion models [121, 122]. OPC depletion during lipopolysaccharide (LPS)‐induced inflammation exacerbates pro‐inflammatory cytokine expression and microglial activation, leading to enhanced neuronal damage, highlighting OPC's protective role in this context [123].

Conversely, in MS and chronic inflammation, OPC immunomodulation may be detrimental. Early in EAE OPCs may exert protective effects by expressing immune checkpoint molecules such as programmed cell death ligand 1 (PD‐L1 or Cd274) [96, 108, 116], which inhibits T cell activation and induces tolerance (reviewed in [124]) (Figure 2A), limiting T cell‐mediated damage in the CNS. However, in EAE or MS, where OPCs are prolongedly exposed to pro‐inflammatory cytokines they acquire an immune or disease‐like phenotype characterized by the expression of antigen‐presenting molecules and other inflammatory cues that may contribute to activate the immune system and propagate inflammation and myelin damage [108, 109, 116, 125]. In vitro IFN‐γ treatment does not only inhibit OPC differentiation [126], but also drives the upregulation of MHCII expression and the phagocytosis and presentation of myelin debris by OPCs to T cells, activating CD4 + T cells in co‐culture and thus, contributing to CD4 + T cell pro‐inflammatory response [108]. Similarly, IFN‐γ exposure also induces MHC‐I expression, enabling OPCs to present antigens to CD8 + T cells, which then secrete IFN‐γ and TNF‐α further contributing to a pro‐inflammatory environment [96] (Figure 2B). Besides activating T cells in vitro, the upregulation of these immune‐related genes appears to also influence disease progression in vivo. OPC depletion through diphtheria toxin expression under the promoter of *Pdgfrα*, a canonical OPC marker, reduces EAE severity and myelin damage [127], implicating OPCs in sustaining chronic inflammation. Although the mechanisms underlying this process are yet to be elucidated, a few key molecular mechanisms have already been identified. Low‐density lipoprotein receptor‐related protein 1 (LRP1), a receptor mediating antigen uptake and endocytosis appears critical: OPC‐specific LRP1 knockout improves outcomes in EAE and cuprizone models without affecting OPC differentiation in vitro [128]. Wnt signaling, essential for regulating OPC proliferation, differentiation and angiogenesis [129, 130, 131], is also implicated in this process; CD4 + T‐cell infiltration into the CNS hyperactivates Wnt in OPCs, accelerating EAE progression through enhanced immune cell recruitment [127].

Based on the above, OPCs are no longer viewed as passive recipients of immune signals, but as active participants in neuroinflammation. Evidence indicates the OPC immunomodulatory role is highly context‐dependent: OPCs can dampen inflammation during acute insults such as LPS exposure, protecting the CNS from further damage [123] but also amplify inflammatory responses under chronic conditions like EAE, worsening disease progression [127, 128]. Despite these insights, the contributions of DA‐OPCs in inflammation and remyelination remain unclear, as does the balance between OPC immunomodulatory and regenerative functions in MS. It is plausible that OPC immune functions are essential for their capacity to differentiate and remyelinate in the inflamed CNS, as suggested by Zveik et al. [106]. Conversely, it is also possible that these immune functions may compete with OPC regenerative capacity, particularly during prolonged inflammation. This complexity underscores the need for combinatorial therapeutic strategies (see Table 1) that simultaneously harness OPC regenerative and immune modulatory potential to preserve axonal integrity and prevent irreversible disability in MS.

## 4 The Perivascular Area: Pericytes as Modulators of Remyelination and Neuroinflammation in MS

PCs represent a subpopulation of platelet derived growth factor receptor beta (PDGFRβ)‐expressing PVCs in the CNS. As previously mentioned, PVCs represent a large and heterogeneous population of cells that also includes vSMCs and PVFs [14, 18, 132, 133]. While vSMCs and PVFs localize in arteries, arterioles, venules and veins, PCs are restricted to microvessels (capillaries) [18, 19, 20]. Notably, PVFs are not the only type of fibroblast that expresses PDGFRβ within the CNS; fibroblasts located in the meninges (dura, arachnoid and pia) also express this receptor. Interestingly, there is a population of PVFs forming a continuum from the pia to infiltrated parenchymal vessels [133]. This population consists of pial/parenchymal PVFs and may detach from blood vessels to penetrate the CNS stroma during neuroinflammation [14].

While there is some knowledge about the role of PVCs in the CNS, it remains somewhat elusive. The current understanding of PCs and their functions in the CNS is limited. Studies have shown that PCs, besides other functions, stabilize the BBB, regulate endothelial cell function and angiogenesis, and modulate capillary blood flow (reviewed in [134, 135]). However, the physiological and pathophysiological roles of PCs beyond vascular function have been scarcely explored. During the last decade the accumulated evidence indicates that PCs may contribute to neuroinflammation, remyelination and its failure in MS. Indeed, active remyelinating MS lesions contain high numbers of proliferative PDGFRβ+ PVCs, compared to non‐remyelinating chronic lesions [34]. Previous studies have explored the role of PCs in MS, particularly addressing their contribution to remyelination and neuroinflammation. In a previous study authors found that early after demyelination PDGFRβ‐expressing PCs proliferate close to differentiating OPCs [23]. After inducing a demyelinating lesion in an animal model that displays reduced numbers of PCs, authors reported a significant delay in OPC differentiation. Further experiments revealed that PCs modulate the regenerative niche by the secretion of Laminin alpha2‐chain (Lama2) that promotes oligodendrocyte differentiation [23]. Moreover, another study showed that soluble factors derived from PCs promote oligodendrocyte fate choice and differentiation in hippocampus‐derived adult NSPCs in vitro [136]. The contribution of PCs to myelin repair is not restricted to the direct modulation of OPC function as it can also do it indirectly. It has been shown that growth hormone receptor pathway in PCs is enough to modulate angiogenesis which indirectly favors OPC differentiation and myelin sheath formation [137]. In addition to remyelination, PCs also participate in neuroinflammation in MS. Indeed, there is evidence showing that PC‐immune cells interactions are essential for BBB permeability and CNS inflammation during MS [138, 139, 140]. When inducing EAE in a PC‐deficiency animal model authors observed an increase in the number of brain‐infiltrating immune cells as well as an increase in disease severity [138]. Interestingly, a reduction in leukocyte infiltration and EAE symptoms was observed following the administration of anti‐VCAM‐1 and anti‐ICAM‐1 antibodies in PC‐deficiency mice, suggesting that the presence of PCs prevents the endothelium from acquiring a pro‐inflammatory profile and thereby protects the CNS from an exacerbated neuroinflammatory response in EAE [138]. A subsequent study reinforced the concept that PCs control vascular immune homeostasis in MS [139]. In this study, authors demonstrated that during EAE, PCs elongate in an effort to restore capillary coverage and BBB stability. However, when exposed to chondroitin sulfate proteoglycans (CSPGs)‐a type of extracellular matrix molecules found in MS lesions‐PCs acquire an “inflamed” phenotype, facilitating macrophage migration and CNS infiltration during EAE [139]. Therefore, PCs abnormalities may lead to changes in CNS vasculature contributing to neuroinflammation in MS. Moreover, a different study explored the capability of PCs to present antigen to T cells and modulate their function [140]. Thus, authors showed in vitro that PCs present antigen and induce T cell activation as well as proliferation [140]. However, how this PC‐T cell interaction contributes to EAE/MS pathophysiology remains unclear.

In summary, PCs' roles extend beyond vascular function, as these cells impact remyelination and neuroinflammation in MS. The underlying molecular mechanisms by which PCs exert their role in MS are still somewhat unknown, as is the contribution of other subpopulations of PVCs, such as vSMCs or PFVs, to MS pathophysiology.

## 5 Blood Elements: Circulating Platelets as Essential Drivers of Neuroinflammation, Neurodegeneration and CNS Repair in MS

Platelets (also known as thrombocytes) are anucleate fragments of cells that are shed from megakaryocytes, located in the bone marrow, to the circulation in very high numbers every day. They remain in circulation for 5 to 9 days (in mice and humans, respectively); with “old” platelets removed from the system in the spleen and the liver [141]. Their main function is to detect areas of vascular damage, in which case they become activated and initiate the formation of thrombi (or blood clots). To execute this function, they constantly patrol the walls of blood vessels [142]; hence, they are a putative key player in the NVN [143]. The detection of areas of vascular damage, but also of pathogen invasion and of cancer, by platelets is mediated by exposure to the basal lamina (an extracellular matrix structure composed of laminins, fibronectin and collagen) and by the interaction with endothelial cells and white blood cells [142]. The ensuing activation of platelets is accompanied by changes in their morphology, the expression of receptors at their surface and the release of a plethora of factors that are stored in granules and in their cytoplasm [144, 145, 146].

**FIGURE 3:**
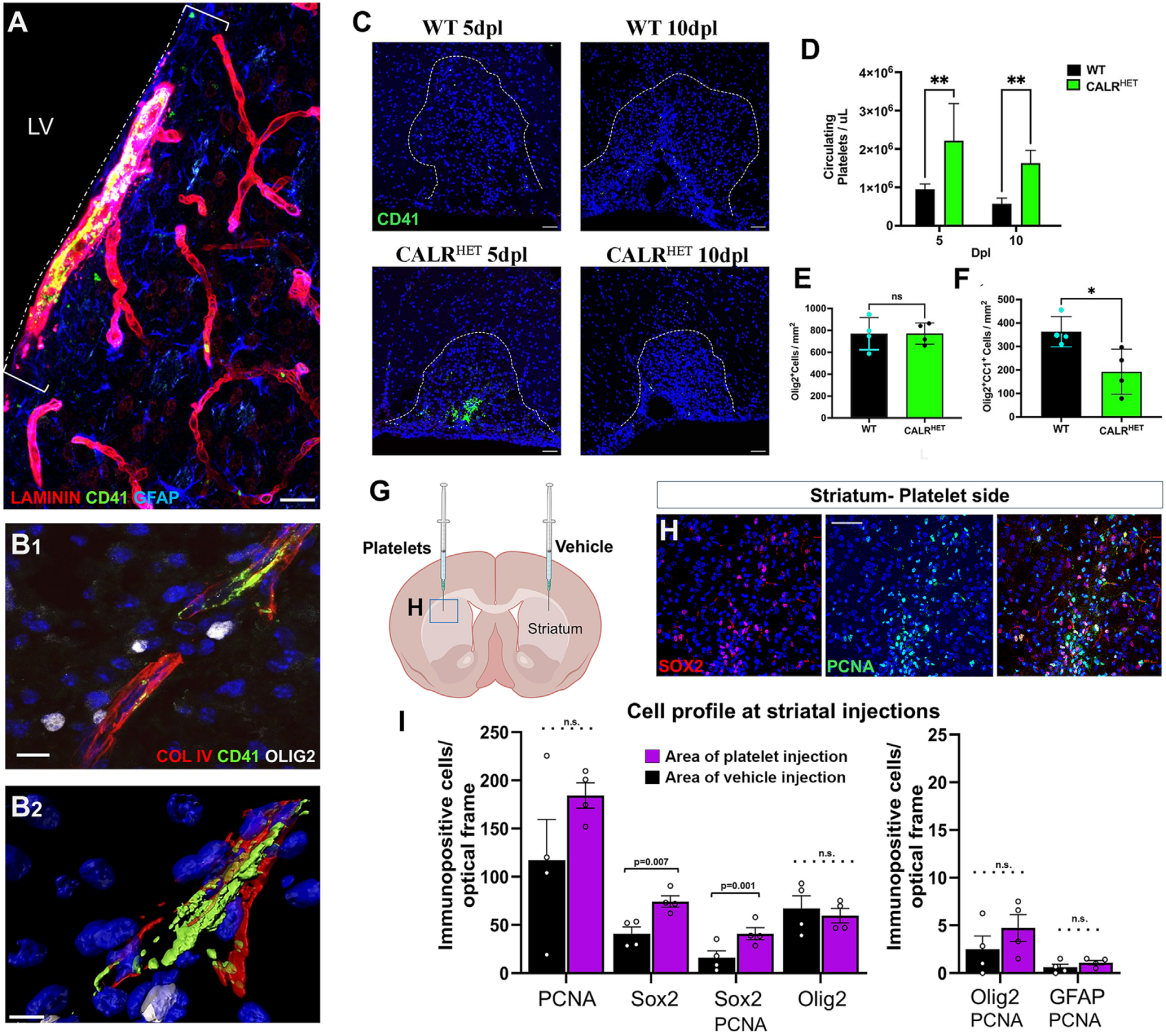
A possible role of platelets as regulators of CNS stem and progenitor cells. (A) Platelets (marked by the expression of CD41, in green) are found to aggregate within blood vessels (marked by laminin expression, in red) within the SEZ neural stem cell niche (the area indicated by brackets, adjacent to the wall of the lateral ventricle (LV)) in response to demyelination in the corpus callosum (area not shown in the image). This is a microphotograph of a mouse brain section after immunohistochemical staining. (B1‐2) CD41+ platelets (in green) are found inside and outside blood vessels (marked by the expression of collagen IV, in red) and near Olig2+ oligodendroglial lineage cells (in white), at the site of focal demyelination in the spinal cord. (C) Immunohistochemical staining of spinal cord tissue, for CD41+ platelets (in green), at the site of focal demyelination in wild type (WT) and *Calr Heterozygous* mice at 5‐ and 10‐days post‐lesion (dpl), reveals increased presence of platelets in *Calr* ^*Het*^ mice. (D) Graph showing that in *Calr* ^*Het*^ mice the number of circulating platelets is significantly increased compared to WT mice. (E) Graph showing that the presence of increased numbers of platelets in the area of demyelination, in *Calr* ^*Het*^, does not affect the density of total Olig2+, oligodendroglial lineage cells. (F) Graph showing that the density of CC1 + Olig2+, myelin‐forming, cells within an area of demyelination in the spinal cord is significantly reduced in *Calr* ^*Het*^ mice. (G) Platelets, or vehicle, were unilaterally injected, directly within the striatum of adult mice. (H) Immunohistochemical staining for Sox2+ neural progenitors (in red) and PCNA+ proliferating cells (in green), at the site of platelet injection in the striatum. (I) Graph showing the numbers of different cell types at the sites of platelet or Vehicle injections. Note the statistically significant increase in the numbers of Sox2+ and of double Sox2 + PCNA+ cells where platelets were injected. [Scale bars: 30 μm in (A), 5 μm in (B), 100 μm in (C), 50 μm in (H). Panels (B)–(F) are adjusted from [33]. Panels (H)–(I) are reproduced by [154]].

Accumulating experimental evidence indicates that besides orchestrating the formation of clots, platelets are intricately involved in the modulation of inflammatory responses [147]; therefore, they have a key role in the processes of tissue degeneration and regeneration [148]. This has led to a recent surge in the investigation of platelets as potential mediators of neuroinflammation and neuroregeneration, having demonstrated that platelet factors [149], or platelets directly injected intracerebroventricularly [150], can affect repair in the nervous system. Parallel work, using transcriptomic and proteomic analyses to investigate aging, led to the identification of Platelet Factor‐4 as a potent regulator of neurogenesis in the hippocampal niche and as a brain rejuvenating factor [151, 152, 153]. Despite these intriguing observations, though, the exact role of platelets in neuropathology and neurorepair remains largely elusive. How often are platelets found in the CNS parenchyma, if at all, in physiological and pathological conditions, and what is their role when this happens? Previous experimental work has shown that platelets aggregate selectively within the vasculature of the SEZ in response to a lesion in the proximal corpus callosum (Figure 3A) [155]. The causes of this aggregation have not been identified, but the niche's vasculature is characterized by slow blood flow [156] and increased levels of leakage [157] when compared to other neighboring areas. The report also did not investigate the results of the aggregation; albeit it was correlated to reduced levels of apoptosis in the niche. Notably, the role of platelets and platelet‐derived molecules in OPC function during remyelination has been explored ([33] and reviewed in [158]). By using chemical ablation strategies to decrease the numbers of circulating platelets in mice, researchers were able to demonstrate that platelets are necessary for efficient remyelination in the spinal cord [33]. This study revealed a bimodal mode of action: while transient exposure to platelets supports OPC differentiation, sustained exposure to these anucleated cells suppresses this effect. In consistency, this study also made an intriguing observation: the presence of increased densities of platelets in areas of demyelination also impaired remyelination (Figure 3B–F). These results, complemented by in vitro experimental work [33, 154, 155] strongly suggest that platelets can affect directly CNS stem and progenitor cells in a dose‐ and time‐dependent manner; an observation reminiscent of neuroinflammatory processes [159]. Using different approaches, recent experimental work showed that the direct injection of non‐activated platelets in the mouse brain parenchyma induces the emergence of mitotic NSPCs, expressing Sox2 (Figure 3G–I), and that the extravasation of platelets in the SEZ leads to increased numbers of OPCs [154]. The presence of platelets in areas of lesion in MS patients has been documented previously [36, 160] and platelets were shown to infiltrate the brain parenchyma and to interact with astrocytes in a mouse model of Alzheimer's disease [161], as well as to constrain amyloid‐related pathology [162]. Therefore, the available evidence strongly indicates a role for platelets at the NVN and within the parenchyma, although the underlying mechanisms and the key modulating factors (tissue infiltration, platelet density, duration of activity) have to be investigated further.

## 6 MS Therapies

For decades, many medical approaches have been developed (and others are currently in development) for the treatment of MS, and numerous target components of the NVN (see details in Table 1). The first drug therapy specifically approved for the treatment of MS dates from 1993: subcutaneously injected Interferon‐β‐1b (IFN‐β‐1b), as a modifier of RR‐MS (for a review in perspective of this treatment, see [50]). Three decades later, there are more than 20 disease‐modifying treatments approved for MS, particularly for the RR‐MS form, including injectable, oral and intravenous immunomodulators, such as ocrelizumab, natalizumab, fenebrutinib, cladribine, ofatumumab, alemtuzumab, dimethyl fumarate and teriflunomide (for currently commercialized, see column 2 in Table 1). These facilitate the control of relapses and the slow‐down of disease progression, but fail to limit neurodegeneration, especially in the progressive forms for which therapeutic options remain very limited [40, 163, 164, 165, 166]. A major reason for this failure, even for fenebrutinib (the Bruton's tyrosine kinase inhibitor), one of the most advanced in the pipeline drugs [167] or for the promising, anti‐histaminic, clemastine [168] (see “neuroprotection” or “remyelinating” in Table 1, column 3), is that they do not enhance the differentiation of OPCs towards mature myelinating cells, which is a key blocking step in the pathobiological mechanism in MS [169, 170, 171, 172, 173, 174]. Given that the most effective drugs are immunomodulators that are administered intravenously and carry a high risk of adverse effects, such as intracerebral bleeding, it is priority to develop strategies targeting OPC differentiation and remyelination capacity, as this will provide neuroprotection and functional restoration [40, 74]. Importantly, even the partial replacement of lost myelin can provide very effective neuroprotection [175, 176, 177].

One major reason behind the lack of success of clinical trials on remyelinating compounds is the existence of relatively poor preclinical studies (Table 1). This stems from the widespread under‐investigation of OPC heterogeneity (determined by the ontogeny and the phylogeny of experimental animal species, the gender, the age, and the overall health condition of the animals) [178, 179, 180]. Another significant reason is the lack of strong preclinical studies with the use of adult human OPCs. Rather, preclinical studies rely on rodent or human cell line OPCs, which differ significantly from the patients' OPCs [163, 166, 181, 182].

Renewed hope has emerged by recent studies using small molecules and aptamers to stimulate remyelination [71, 74, 77, 183] (Table 1). In both compound cases, the drugs seem to act both as remyelinating and anti‐inflammatory agents, in agreement with the recent evidence highlighting the interplay between inflammation and remyelination in MS [106, 184]. They also take advantage of the reduced immunological complications of these molecules, as compared to the use of monoclonal antibodies [185].

## 7 Conclusions

MS is a progressive and incapacitating disease that has been vested with high hopes for the development of effective treatments, mainly because it involves the gradual, age‐correlated, derailment of a regenerative process that is robust in the human brain. The inflammatory aspect of the disease, which is responsible for the loss of myelin, has been extensively investigated and elucidated, hence leading to the available interventions (see Sections 2 and 6 of this review) that have led to significant success in slowing down the progress of the disease and in blocking further relapses. However, the identification of the mechanisms that result in the failure of the remyelinating capacity of OPCs remains elusive. Here, we summarized the growing evidence on the key role the NVN is playing in the healthy and the pathological CNS. Thanks to the rapid development of high‐throughput methodologies, the heterogeneity of OPCs is intensely investigated and has shed light to novel functions, such as their ability to actively modify neuroinflammation (see Section 3). Strikingly, recent evidence revealed that OPCs bear primary cilia, that is, cellular components that are known to facilitate key signaling processes in neural stem cells, and to exert phagocytosis behavior that contributes to axonal pruning and reshaping [186]. Besides the OPC aspect, the development of spatial transcriptomic, as well as of multi‐omic, technologies start to bring together gene‐expression and anatomical analyses [98], an approach of special interest for the further investigation of the role and of the response of the NVN, which is an architecturally complicated and diverse structure, in MS. Another result of the extensive application of RNAseq approaches is the identification of the heterogeneity of different cell types, including OPCs, but also ‐more recently‐ of oligodendrocytes. It now emerges that subsets of oligodendrocytes respond differently to the disease [187] and these observations are likely to inform pre‐clinical and clinical work aiming at the development of treatments. The presence of oligodendrocytes within, or very proximal, to the NVN [188] will inevitably lead to the necessity to investigate if specific oligodendrocyte subtypes behave in different ways in response to pathological changes of the NVN. Moreover, the existence of differences in the properties/behavior of OPCs [189] and of oligodendrocytes [190] according to their anatomical location (e.g., in the white or the gray matter) also begs the question of the existence of differences in the NVN according to their location with the CNS. Finally, the identification of all the cellular players that form, and are active, within the NVN (see chapters 1, 4 and 5), again in combination with the continuous generation of multi‐omic data from experimental models and patients, might prove valuable in our ability to stratify MS patients, aiming at the development of more personalized interventions [191].

## Author Contributions

**Francisco J. Rivera:** conceptualization, writing – original draft, writing – review and editing, visualization. **Maria Elena Silva:** conceptualization, writing – original draft, writing – review and editing, visualization. **Fernando de Castro:** conceptualization, writing – original draft, writing – review and editing, visualization. **Alerie G. de la Fuente:** conceptualization, writing – original draft, writing – review and editing, visualization. **Ilias Kazanis:** conceptualization, writing – original draft, writing – review and editing, visualization.

## Funding

This work was supported by the Research Council of Finland (Suomen Akatemia) ‐ Academy Project 370013 to Francisco J. Rivera; the Sigrid Jusélius Foundation (Sigrid Juséliuksen Säätiö) to Francisco J. Rivera; the Finnish Multiple Sclerosis Foundation (Suomen MS‐Säätiö) to Francisco J. Rivera; and the Chilean National Agency for Research and Development (Agencia Nacional de Investigación y Desarrollo, ANID) ‐ FONDECYT Regular 1201706 to Francisco J. Rivera. In addition to this, this work was supported by the Spanish Ministerio de Ciencia e Investigación‐MCIN and the Agencia Estatal de Investigación‐AEI (grant PID2022‐143110OB‐I00 funded by MCIN/AEI/10.13039/501100011033/and by “ERDF A way of making Europe”, by the European Union to Fernando de Castro and PID2024‐161979OB‐I00 funded by MICIU/AEI/13.13039/501100011033 and EDRF/EU to Alerie G. de la Fuente), a contract #050601240019 “LeukoReMy” (financed by Asociación Europea contra la Leucodistrofia‐ELA ‐Spain‐ and Fundación Hesperia ‐Spain‐) to Fernando de Castro and a Ramón y Cajal Fellowship from the Ministerio de Ciencia Inovación y Universidades, Agencia Estatal de Investigación (RYC2023‐045776‐I, funded by MICIU/AEI/13.13039/501100011033 and FSE+) to Alerie G. de la Fuente. Also, by a research grant from the Hellenic Foundation for Research and Innovation (HFRI‐FM17‐3395) to Ilias Kazanis.

## Conflicts of Interest

The authors declare no conflicts of interest.

## Data Availability

Data sharing not applicable to this article as no datasets were generated or analysed during the current study.
